# Supplementary material for: Some animals are more equal than others: Validation of a new scale to measure how attitudes to animals depend on species and human purpose of use
Source: PLoS One. 2020 Jan 21;15(1):e0227948. doi: 10.1371/journal.pone.0227948 (PMC6974055; doi:10.1371/journal.pone.0227948)
Supplement: S1 Appendix — The question ‘How do you feel about the use of…’ was repeated in the identical format for 10 types of animal. Thus participants were asked to repeat their ratings also for rats, rabbits, pigs, monkeys, octopus, chickens, badgers, zebrafish and tree shrews (just the animal to be considered was changed in the question line). (DOCX) [file pone.0227948.s001.docx]

**Animal Purpose Questionnaire (APQ)**

Please rate whether you agree or disagree with the killing of different types of animal for the following purposes:

- medical research (of any kind e.g. for an animal model of dementia)
- basic science (of any kind e.g. to better understand the brain)
- food production (of any kind e.g. bush meat in the tropics where applicable)
- pest control (of any kind e.g. when crops are damaged)
- other cultural practices (e.g. the use of body parts as ornaments)

Tick the appropriate box on the rating scales to indicate your level of agreement of disagreement with any kind of use, within each of these broad categories, which directly or indirectly results in the killing of the animal.

Strongly Disagree = 1, Disagree = 2, Neutral = 3, Agree = 4, Strongly Agree = 5.

Not all animals included in the field of question are used or have ever been used in the UK, please rate your agreement/disagreement for comparison.

1. How do you feel about the use of **mice** for the following purposes:

**Medical research**

| Strongly Disagree | Disagree | Neutral | Agree | Strongly Agree | Not applicable |
| --- | --- | --- | --- | --- | --- |

**Basic science research**

| Strongly Disagree | Disagree | Neutral | Agree | Strongly Agree | Not applicable |
| --- | --- | --- | --- | --- | --- |

**Food production**

| Strongly Disagree | Disagree | Neutral | Agree | Strongly Agree | Not applicable |
| --- | --- | --- | --- | --- | --- |

**Pest control**

| Strongly Disagree | Disagree | Neutral | Agree | Strongly Agree | Not applicable |
| --- | --- | --- | --- | --- | --- |

**Cultural practices**

| Strongly Disagree | Disagree | Neutral | Agree | Strongly Agree | Not applicable |
| --- | --- | --- | --- | --- | --- |

**Appendix 1.** The presentation format used for the animal purpose questionnaire (APQ) in survey 1. The question ‘How do you feel about the use of…’ was repeated in the identical format for 10 types of animal. Thus participants were asked to repeat their ratings also for **rats**, **rabbits**, **pigs**, **monkeys**, **octopus**, **chickens**, **badgers**, **zebrafish** and **tree shrews** (just the animal to be considered was changed in the question line).
