# Supplementary material for: Some animals are more equal than others: Validation of a new scale to measure how attitudes to animals depend on species and human purpose of use
Source: PLoS One. 2020 Jan 21;15(1):e0227948. doi: 10.1371/journal.pone.0227948 (PMC6974055; doi:10.1371/journal.pone.0227948)
Supplement: S2 Appendix — The question ‘To what extent do you agree with the use of…’ was repeated in the identical format for the 5 categories of purpose. Thus participants were asked to repeat the ratings also for BASIC SCIENCE RESEARCH, FOOD PRODUCTION, PEST CONTROL and OTHER PRACTICES. (DOCX) [file pone.0227948.s002.docx]

You will now be asked to rate whether you agree or disagree with the killing of different types of animal for the following purposes:

**medical research** of any kind e.g. for an animal model of dementia

**basic science research** of any kind e.g. to better understand the brain

**food production** e.g. any form of commercial or domestic consumption of animal product

**pest control** removal of an animal that is impacting adversely on human activity e.g eradicating a 'pest' if it had damaged crops or invaded homes ​

**other uses** e.g. for hunting or fighting the animals as a sport, for wearing skin as fashion or as ornamentation and displaying the body as a trophy

Select the appropriate option on the rating scales to indicate your level of agreement or disagreement with the use or treatment of animals, within each of these broad categories, which directly or indirectly results in the killing of the animal.

Even if the use or treatment of the particular animal seems unlikely, imagine it was a typical use of the animal and make the judgement accordingly. 

- **Check here to continue**

To what extent do you agree with the following animals being used for **MEDICAL RESEARCH?**

|  | Strongly Agree (1) | Agree (2) | Neutral (3) | Disagree (4) | Strongly Disagree (5) |
| --- | --- | --- | --- | --- | --- |
| Pig (1) |  |  |  |  |  |
| Chicken (2) |  |  |  |  |  |
| Dog (3) |  |  |  |  |  |
| Dolphin (4) |  |  |  |  |  |
| Chimpanzee (5) |  |  |  |  |  |
| Rabbit (6) |  |  |  |  |  |
| Rat (7) |  |  |  |  |  |
| Snake (8) |  |  |  |  |  |
| Frog (9) |  |  |  |  |  |
| Pigeon (10) |  |  |  |  |  |
| Carp (fish) (12) |  |  |  |  |  |
| Parrot (13) |  |  |  |  |  |

**Appendix 2.** The Qualtrics presentation format for the animal purpose questionnaire (APQ) in survey 2. The question ‘To what extent do you agree with the use of…’ was repeated in the identical format for the 5 categories of purpose. Thus participants were asked to repeat the ratings also for **basic science research**, **food production**, **pest control** and **other practices**.
