# Supplementary material for: Some animals are more equal than others: Validation of a new scale to measure how attitudes to animals depend on species and human purpose of use
Source: PLoS One. 2020 Jan 21;15(1):e0227948. doi: 10.1371/journal.pone.0227948 (PMC6974055; doi:10.1371/journal.pone.0227948)
Supplement: Attachment — Submitted filename: PONE responses to reviews.docx [file pone.0227948.s005.docx]

**PONE-D-17-40454: Responses to original reviewers**

Original submission: Some animals are more equal than others: Attitudes to animals in relation to species and human purpose of use.

Revised manuscript: Some animals are more equal than others: Validation of a new scale to measure how attitudes to animals depend on species and human purpose of use.

**We now submit a substantially revised manuscript which includes further data collected using an online version of the survey. Further specific points raised by the referees have also been attended to as detailed below.**

**Reviewer #1:**

In summary, the reviewer agreed that the APQ has the potential to be a useful measure of beliefs about the use of animals, but judged the original paper (which reported the results of a single paper-based survey) to be unready for publication in PLoS without the results of an online survey method, to increase the sample size and diversity.

Abstract

The abstract should include information about the number and nationality of the subjects.

**The surveys were completed by 415 British nationals and 68 participants from 39 other countries. This information has been included in the revised abstract.**

Introduction

The introduction is well-written, and it offers a good review of the literature. The second paragraph discusses incongruities in animal protection legislation. The authors may want to mention a particularly egregious example - the Animal Welfare Act in the United States, which specifies that rats, mice, and birds are not animals under the legislation.

**The US Animal Welfare Act is now mentioned p3 para 2.**

Page 6, line 12. “Pet, pets, and profit” The author cite Taylor and Signal’s paper using their Pets, Pets, and Profit (PPP) Scale. However, the manuscript does not mention their scale. Like the APQ, the PPP is also a multi-dimensional alternative to the Animal Attitude Scale.

**The relevance of the PPP scale has been clarified p6 and its importance further acknowledged in the Discussion p18.**

Page 6, line 16. “Risk to life” The risk to life items were included to compare attitudes to the treatment of two animals (dogs and sharks) with attitudes to the treatment of humans. But for reasons described below, I suggest dropping these items in future administrations of the scale.

**As suggested the ‘risk to life’ items have been dropped.**

Methods

Subjects

The sample included only 39 males and 89 females; increasing the number of subjects would make the results more robust. In addition, the generalizability of the results is limited as all the subjects came from the University of Nottingham. Further, over half of them were first-year psychology students. An better alternative would be to recruit a larger and more diverse sample using an internet data pool such as Amazon’s MTurk.

**With the use of a follow-up online Qualtrics survey, we have now increased the sample size to 483. The online survey also attracted more female than male participants but the absolute number of males is now 144, allowing for a more robust consideration of the sex difference. Moreover the online survey was widely circulated, including in Malaysia. Thus although the sample was still on the whole highly educated, its cultural and ethnic diversity has been improved. There was no financial incentive to participate in the present study and the use of MTurk would not necessarily result in a truly representative sample either (see e.g. Bohannon, J., S*cience* 21 Oct 2011: Vol. 334, Issue 6054, pp. 307; Singal, J., *The Cut*: Social Psychology 9 June 2016).**

More information on the subjects is needed. For example, how was “diet” assessed and how many subjects were in the various dietary groups?”

**Diet was assessed by self-report in response to a question in the demographics section of the survey – participants were requested to select the descriptor that best matched their diet. The breakdown of the samples by dietary group and survey is reported in Table 1, in percentage format for comparison across the surveys.**

Questionnaire

The description of the APQ is inadequate. The statement that “some examples” were used to illustrate each animal use purpose is unclear. How many examples were given for each purpose? What was the specific wording of the items? Is bush meat really a good example of the use of animals for food production? Did all of the subjects really know what bush meat is? The category “other cultural practices” is ambiguous.

**The examples were given in the format described p9 for survey 1 (as examples). The qualifier ‘some’ has been deleted in the revision. The wording of the examples used to illustrate the different purposes of use was improved for the online version of the questionnaire, as described p10 for survey 2.**


The statement “as per the AAS” is misleading as the AAS is scored in the opposite direction as the APQ, (High scores on the AAS indicate pro-animal welfare attitudes.)

**This has been removed in the revision.**

I did not understand the instruction that subjects were to extrapolate to “other uses of any kind.”

**This has been rephrased p9.**

It might help if this section were clearly divided into a “purpose of animal use” section and an “activities which risk life” section. The risk to life items were evaluated with three species – sharks, dogs, and humans. I found these items baffling. The strange pattern of results in Table 2 suggests the subjects also had trouble understanding these items. I suspect that many of them indicated that the items were “not applicable,” in which case they were automatically assigned a score of 2.99. This issue might be clarified if the actual items on the scale were included in the manuscript.

**The risk to life items have been removed.**

Procedure

The authors state that the survey could not be given on-line because it was necessary for the experimenters to explain items and define terms to the subjects. This suggests that the some of the items/instructions were confusing. In addition, researchers are increasingly conducting human-animal interaction questionnaire studies via the Internet. Thus the need to have an experimenter present to explain the APQ is a major problem if the authors intend for the scale to be used by other investigators.

**We now include the results obtained using an online version of the questionnaire.**

Results

Page 10, last paragraph. Here, as in several other places in the results section, large numbers of descriptive statistics are embedded in the text. This makes for difficult reading, especially for making comparisons between species.

**The results of survey 1 are now presented in a more focused format and the figures and tables are combined into panels, to facilitate comparison across species and purpose of use (as well as across the survey formats).**

Page 15. The finding that there were no sex differences in diet is interesting and surprising. Most studies have found that women have higher rates of meat avoidance than men. However, it is unclear how diet was assessed, and the number of men and women in various categories (vegan, vegetarian, omnivore, frequency of meat-eating, etc.) is not reported.

**The results of survey 2 (which had a larger N and included 105 males) showed a positive association between diet and sex: males were more likely to report eating meat. The revised Table 1 reports the breakdown of the samples by dietary group, in percentage format for comparison across the surveys.**

Discussion

Page 18, line 4. Cite the Taylor and Signal PPP scale here?

**The Taylor and Signal PPP scale is now cited as suggested.**

Page 19, last paragraph. This is a good use of responses to the open ended item.

**We’ve retained the use of open-ended responses to clarify participants’ reservations about the use of animals in medical research.**

The term “self-reported gender” is awkward. We would not say “self-reported pet owners” or “self-reported first year college students.”

**The term ‘self-reported’ has been removed as suggested.**


Reviewer #2:

Although alternative to methods to gauge attitudes to animals already exist, this is arguably a valuable contribution. However, methodological weaknesses and structural deficiencies should be addressed.

Specific Comments

Title – The title is appealing but should reflect more prominently the validation/development of a new method for measuring attitudes to animals.

**The title has been amended as suggested to focus on the validation aspect of the study.**

Introduction – The section requires restructuring. The introduction is arguably excessively long and some of its contents could be moved to the discussion. Alternatively, sub-headings should be used. An accepted definition of speciesism is missing. Moreover, it is not enough for a legislation to protect some species more than others to call it speciesist. There are good reasons why dogs, and not ants, are protected by law; it must be demonstrated that current legislation discriminates against certain animal species (for frivolous reasons). The two last paragraphs present the APQ and therefore should belong to the methodology section.

**As suggested the Introduction has been restructured with subheadings. The misuse of the term speciesism has been corrected and speciesism has been defined p4. Minimal details of the APQ are presented in the Intro, just by way of introducing the present study.**

Methodology –

I had a hard time understanding the second sentence from the first paragraph and I was not convinced about it usefulness. Please rephrase or remove.

**This sentence has been removed as suggested.**

Most of the section ‘participants’ describes demographic results and therefore should be part of the results section.

**I tried relocating this information to the Results but it didn’t sit right and the size and diversity of the samples are related to the sampling strategy in use. In the revised manuscript, there are separate Results sections (for each of the surveys) exploring the interrelationships between questionnaire scores and demographic factors. We also have suggested that the Table 1 summary of the demographic information be positioned early within the Results section, to reduce the appearance of result-like information in the Methods.**

Additionally, there is no rationale explaining the choice of participants. What guided de sampling procedure? Moreover, some concerns arise regarding study procedures: bearing in mind that psychology students received class credit for participation, what measures were in place to avoid social desirability bias? You mention that experimenters followed standardised procedures but you fail to mention if participants knew or not the experimenter.
I was not elucidated on the age distribution in both sub-populations (students and mixed). The proportion between men and women each subpopulation is also not described.
The structure of the new APQ is not described in sufficient detail. Please include the APQ as annex. According to the journal’s ethos, the authors are required to make all data underlying the findings described in their manuscript fully available without restriction. I also was not convinced by your justification to equal ‘not applicable’ to neutral. Does this mean that participants were prevented from not responding?

**The reviewers’ further specific concerns are in effect addressed by the inclusion of the results of a second survey, conducted with some improvements to the item wordings and scoring, and conducted online using Qualtrics to reach a larger more diverse sample. Despite the deficiencies of survey 1, survey 2 yielded closely similar results. The available demographic details have been provided in Table 1. The APQ has been included as an annex. Both of the data files have been submitted together with the manuscript and will be placed in our institutional data repository when the findings we wish to report are accepted for publication.**

**For the APQ in survey 1 (only), a ‘not applicable’ choice option was provided and scored 2.99 (equivalent to the median rating of neutral) in order that the participants’ other responses were not excluded (because of the repeated measures nature of the design used to test for interactions between species and purpose of use). This ‘not applicable’ response option was dropped for the survey 2. In both surveys, participants had the option to withhold responses.**

Discussion
In the section “Effects of purpose of use as measured by the APQ” the authors voice participants opinions (additional comments). These are, in effect, results and should be treated as such. Furthermore, are participants’ comments rich enough to justify a qualitative content analysis that may prevent anecdotalism?

**Unfortunately, we don’t believe we have sufficient open-ended comments for a qualitative analysis. We’ve left these comments in as quotes, in line with one of the comments from Reviewer 1: “Page 19, last paragraph. This is a good use of responses to the open ended item.” We therefore retained the use of open-ended responses, to clarify participants’ reservations about the use of animals in medical research.**

The segment “All the participants completed all of the questionnaires in the same fixed order. Within each questionnaire, the item order was also fixed.” belongs to the methodology.
The statement “Fixed item order is the standard procedure for established questionnaires” requires references.

**This segment has been removed for brevity, and clearly the item order is not an issue as this was randomised in survey 2 as explained in the Methods p9.**

Finally, results should be seen as instrumental towards validating the APQ. The heterogeneous convenience sample of participants is not representative of any population and no extrapolations can be drawn from its results, other than having a new tool for measuring attitudes to animals.

**The title now better reflects the purpose of the study, to validate a new tool for measuring attitudes to anima use. The sampling strategy used for new the online part of the study (survey 2) reached a more diverse sample.**
