# Supplementary material for: Some animals are more equal than others: Validation of a new scale to measure how attitudes to animals depend on species and human purpose of use
Source: PLoS One. 2020 Jan 21;15(1):e0227948. doi: 10.1371/journal.pone.0227948 (PMC6974055; doi:10.1371/journal.pone.0227948)
Supplement: Attachment — Submitted filename: Response to Reviewers APQ PLOS ONE.docx [file pone.0227948.s006.docx]

Ref: PONE-D-19-23588
Title: Some animals are more equal than others: Validation of a new scale to measure how attitudes to animals depend on species and human purpose of use.
Journal: PLOS ONE

**Journal Requirements**

We have checked the PLOS ONE style requirements and labelled the files as per the templates.

Captions for and in text citations of the Supporting Information files have been provided.

**Response to Reviewers**

The checkbox responses are positive with the exception of Q2 (Reviewer #1). The specific points concerning the statistical analysis will be addressed below.

**Reviewer #1:**

This is the second time I have reviewed this manuscript for PLOS ONE. This version is much improved, and the reviewers addressed the issues that I had with the first version.

*We are pleased that the reviewer finds the manuscript much improved and finds the initial issues raised to have been addressed.*

Statistical analysis: I am concerned the lack of information on the reliability of the APQ. It is standard practice to include information on the internal reliability of new scales using, for example, Cronbach’s alpha. The authors mention that the AAS=10 has high reliability (Cronbach’s alpha), but this is based on single scores calculated by summing responses to the 10 AAS items. As far as I could tell, no information is given on the reliabilities of the various subscales of the APQ. This information could be included in a separate table.

*We appreciate that information on reliability might normally be expected to appear in the Methods. However, since it needed to follow on from the factor analysis, the Cronbach’s alphas for the five purpose subscales of the APQ were reported in the Results part of the manuscript (p15 under the subsection of ‘Correlation and regression analyses to explore the interrelationship between questionnaire scores and demographic factors). In addition, we have now added a sentence highlighting the overall reliability of the scales for studies one and two (both α = .98; Study 2: α = .98). We have also added a sentence explaining the reliabilities by animals across purposes for surveys one and two (p20).*

While it is too late now, several of the descriptions of the use categories could be improved. For instance, the term “Other Cultural Practices” covers a lot of territory. The exemplar in the scale is “using body parts as ornaments.” And the text of the paper mentions bullfighting as an example. However, positive uses of animals such as pet-keeping and bird feeding are also “other cultural practices.” Similarly, I am not sure that “bushmeat” is the best example of the use of animals for food.

*The limitation that the examples provided to illustrate the category of other cultural practices were all negative has been acknowledged p29. As explained p11, the wording of the examples given to illustrate the purposes of use was improved in survey 2 (the reference to bush meat was dropped).*

Comparisons between the AAS and the APQ are frequently made in the manuscript. The problem is that the AAS is scored so that individuals who are concerned with animal welfare (that is, generally oppose the use of animals) get high scores. However, the APQ is scored so that individual who generally disapprove various uses of animals and species get low scores. Hence, AAS and APQ scores are highly related – but in in the opposite directions.

*The expected direction of association between the scales has been clarified p12.*

This can create confusion even in the present ms. For example, Lines 467 – 469 state “Diet had a negative effect on AAS scores and all facets of the APQ questionnaire indicating that those who eat meat have less animal welfare concerns and were more willing to use animals.” Yet according to Table 3b, the APQ scores were actually correlated positively with meat-eating – as would be expected.)

*The statement of this result has been clarified (with reference to Table 3) p21.*

For this reason, it might be a good idea to include a paragraph emphasizing that the differences in the scoring of these scales. Even though the scales assess similar underlying dimensions of attitudes toward animals, the APQ is a measure of the degree of approval of various uses of animal by humans while the AAS measures concern for animal used in various situations.

*Apologies for the error, we have now corrected the effects of diet on APQ to be in agreement with Table 3 (p21). We agree with the reviewer’s point that adding a paragraph emphasizing the different scoring of the scales would help with the clarity of the manuscript. This has been done p12.*

Minor suggestions

-When citing papers in Anthrozoos, there is no need to include “A Multidisciplinary Journal of the Interactions of People and Animals” in the citation.

*The citations to Anthrozoos have been corrected throughout the reference list. Other references have also been reformatted to match journal style.*

-The new Speciesism Scale by Caviola et al. covers much of the same territory of the AAS and the APQ. It should be cited and possibly discussed. The reference is Caviola et al (2019) The moral standing of animals: Towards a psychology of speciesism. J Pers Soc Psychol. 116(6), 1011-1029.

*The suggested reference has been cited. The APQ is much simpler in conception, in that we examine attitudes to animal use systematically by species and purpose of use. Since it is any difference in ratings by species and/or purpose of use which is of interest we have an inbuilt control for the demand characteristics of the scale. The Speciesism Scale uses more conventionally formulated items (some modified from existing scales), followed by factor analysis. In contrast, the APQ has the a priori factors of species and purpose. We agree that the Speciesism Scale presents a very valuable addition to the literature and this has been highlighted p30 but the approach taken in the present study is quite different.*

Table 2 - Indicate what the subscale initials mean on the captions (MR, BS, FP etc.) This is done in the other tables.

*The Table 2 abbreviations are highlighted in the revision (they might slip off the page if the format changes in file conversion).*

Animals. I was surprised the lack of variation in some of the species. It might be interesting in future administrations to include, say, fruit flies.

*We agree that the selections of species were arbitrary and certainly not an exhaustive list. In the same way we made some changes to the species selected for surveys 1 and 2 we hope that others will adopt and adapt the proposed questionnaire format to suit their research purposes. We agree that invertebrates have been understudied to date and that this omission should be rectified in future studies. This limitation has been acknowledged p30.*

**Reviewer #2:**

Overall impression - The submitted manuscript describes the development of a new attitude to animal scale in relation to species and purpose of use, the Animal Purpose Questionnaire. Building upon a previous submission, the authors have submitted the APQ to two different populations, together with the established Attitudes to Animals Scale. Despite these efforts, some methodological concerns remain. The introduction is both informative and concise.

*We are pleased that the reviewer finds some improvement in the manuscript, e.g. in the introduction.*

The authors have revised the manuscript by providing further data. There are now results from two different sets of studies: one smaller pen and paper survey to a population mostly made of female, 21 or under, psychology British students (N=128), and a wider on-line survey to 419 participants (most participants were female, most own pets and there were 39 nationalities represented). Both populations were conveniently sampled. Considering that the attitudes to animals are often culturally constructed, the high number of different nationalities may pose a challenge for the interpretation and generalization of results. The low proportion of men and respondents not owning pets are also limiting factors (the authors acknowledge these limitations).

*As discussed p30, we agree that there is further work to be done to test the generality of the findings.*

Although both studies yield comparable results, I am not sure that presenting them together is the best approach. In fact, methodological differences between both surveys render meaningful comparisons problematic. First there is the imprinting effect from the examples. The examples of purpose of use in survey 1 (e.g. bush meat for food production) differed from those in survey 2. In addition, the difference between medical research and basic science may not be entirely clear for most participants (even I was unsure of their remit). Moreover, the changes made to the wordings and scoring in survey 2 must be considered. For example, removing the ‘Not applicable’ option in survey 2 is not without its flaws. Finally, there is the issue of survey mode of administration: pen & paper surveys are known to elicit different response biases than online surveys. I would suggest considering survey 1 as a pilot/development study (and publishing it as such), and survey 2 as the validation study, which has been improved in accordance to the results of survey 1.

*Our justifications to include survey 1 include the demonstrated reproducibility of some of the main findings shown in survey 2 despite the methodological differences (beyond presentation format) identified by the reviewer. The survey 2 data show broadly the same profile of responses across the purposes of use. The effects of sex and diet on the APQ and AAS scores are also consistent across the two surveys. The point of the comparison notwithstanding, these differences have been further acknowledged p30. Survey 1 also shows the viability of administering the questionnaire in paper format, paper format can be preferable for example to assess the effectiveness of outreach activities*.

*We take the point that survey 1 can be referred to as a pilot/development study and survey 2 as the validation study and this terminology has been adopted in the revised manuscript (p7 and elsewhere).*

In terms of results, the large number of variables being calculated raises some concerns regarding p-hacking. For example, for the comparison between AAS/APQ scores and demographic variables, generated 110 correlations alone (Table 3), but not all are equally relevant, and some may even be spurious (e.g. with such a predominantly British population, there is little use of analysing the effect of nationality). This generates some noise in the discussion when trying to explain differences between survey 1 and the more methodologically robust survey 2. Finally, the fact that the use of animals for basic science did not differ significantly from their use for medical research may be no more than a reflection of the lack of power of the APQ in differentiating between the two.

*The point about noise in the discussion has in part been addressed by the revised terminologies for the surveys. The lack of difference seen in attitudes to using animals for basic science vs medical research could reflect lack of power but the statistical power was quite sufficient to detect differences in attitudes in relation to other purposes of use. Even if it’s just a smaller effect size for the difference between medical research and basic science we’d still see that as of interest. Still we have added the suggested caveat p24.*

*We agree that a large number of bivariate correlations raises the risk that some effects may be spurious. We wish to point to a number of key findings which replicated across the surveys, please see also above response to the point made about comparing across the surveys.*

*However, we agree that a note of caution is warranted in the interpretation of the findings so we raise the reviewer’s point in the limitations section (p30).*
